# ASO Author Reflections: Redefining Breast-Conserving Therapy: National Trends in Oncoplastic Reconstruction

**DOI:** 10.1245/s10434-026-19985-4

**Published:** 2026-06-17

**Authors:** Virginia Bailey, Luke Juckett, Naomi Ghahrai, Sydney Pessin, Lynn Dengel, Scott T. Hollenbeck

**Affiliations:** 1 Florida State University College of Medicine, Tallahassee, FL USA; 2 Department of Plastic Surgery, University of Virginia, Charlottesville, VA USA; 3 Virginia Commonwealth University School of Medicine, Richmond, VA USA; 4 Department of Surgery (Surgical Oncology), University of Virginia, Charlottesville, VA USA

## Past

The management of breast cancer has undergone a paradigm shift over the past several decades, largely driven by landmark trials such as the NSABP B-06, which demonstrated equivalent oncologic outcomes between mastectomy and breast-conserving therapy (BCT).^1–4^ These findings established lumpectomy with radiation as a safe and effective alternative to mastectomy and catalyzed a national transition toward breast conservation. Concurrently, advances in surgical technique gave rise to oncoplastic breast surgery (OPS), integrating oncologic resection with reconstructive principles to mitigate contour deformities and improve aesthetic outcomes.^4^ Despite widespread adoption of BCT, national-level data characterizing trends in oncoplastic reconstruction have remained limited, leaving an incomplete understanding of how reconstructive practices have evolved alongside oncologic care.

## Present

In our national claims-based analysis (PearlDiver) of more than 480,000 patients undergoing lumpectomy from 2010 to 2020, we demonstrated a substantial increase in oncoplastic reconstruction, with a 249% increase in lumpectomy with reconstruction procedures over the study period.^5^ This growth was primarily driven by adjacent tissue rearrangement and reduction mammaplasty, reflecting a shift toward more complex reconstructive strategies. These findings parallel broader national trends showing increased utilization of breast-conserving approaches and evolving surgical priorities.^6^ Importantly, this trend underscores a fundamental change in breast cancer care—from a sole focus on oncologic safety to a more holistic emphasis on aesthetic outcomes and quality of life. At present, a few small studies have shown that oncoplastic techniques are associated with improved patient-reported satisfaction, psychosocial well-being, and cosmetic outcomes compared with both standard lumpectomy and mastectomy.^3,4^ Our findings suggest that this concept is being applied increasingly at a population level. However, our analysis also highlights disparities in access to oncoplastic reconstruction, particularly with respect to insurance status and comorbidity burden. Patients undergoing reconstruction were more likely to have commercial insurance, consistent with prior literature demonstrating structural barriers to equitable access in breast cancer care. These disparities emphasize that while surgical innovation is advancing, its benefits are not uniformly distributed.

## Future

As oncoplastic reconstruction becomes increasingly integrated into standard breast-conserving therapy, future efforts must focus on (1) optimizing patient selection, (2) enhancing access to advanced techniques, and (3) critical evaluation of long-term outcomes. Patient selection for oncoplastic approaches remains challenging. Many surgeons are hesitant to perform breast conservation and advanced tissue rearrangement procedures in the setting of large, multifocal, or multicentric tumors. Additionally, tumor location is critically important when developing partial breast replacement surgical approaches. These patient specific factors have not been adequately addressed in most studies, leading readers to assume standard oncoplastic approaches can be applied universally irrespective of the nature of the expected defect. Second, health systems research should address structural barriers; including insurance coverage, referral patterns, both of which could limit equitable access to reconstruction. Finally, future studies promoting oncoplastic approaches should evaluate long-term outcomes, including breast cancer surveillance and local recurrence, revision surgery for fat necrosis and asymmetry, cost-effectiveness, and resource utilization. Ultimately, the evolution of breast cancer management will undoubtedly include oncoplastic surgery principles and techniques. Growing enthusiasm and early adoption must be balanced with defining strategies for optimal patient selection, equitable delivery of services, and critical analysis of long-term complications, including radiation fibrosis, fat necrosis, and local recurrence. Still, it is clear that oncoplastic surgery will play an increasingly important role in managing patients with breast cancer.
